# Acoustic performance analysis of wooden structure building wall by integrating BIM technology and impedance tube method

**DOI:** 10.1371/journal.pone.0308481

**Published:** 2024-08-09

**Authors:** Jia Yin, Xiangren Ai

**Affiliations:** School of Civil Engineering, Liuzhou Institute of Technology, Liuzhou, China; Brno University of Technology Faculty of Civil Engineering: Vysoke uceni technicke v Brne Fakulta stavebni, CZECHIA

## Abstract

With the increasing demand for building acoustic performance, accurately evaluating the acoustic performance of building walls has become an important research topic. However, existing research has mostly focused on general building materials such as concrete, iron and steel, and glass. For wooden structure wall, due to the sound absorption performance of the materials themselves and the complexity of structural design, the analysis of their acoustic performance is still relatively weak. Moreover, there is a lack of quantitative description of their spectral characteristics and acoustic impedance. To analyze the acoustic performance of wooden structure building walls, Building Information Model (BIM) and impedance tube method were integrated to construct a building wall performance testing system with BIM technology. The impedance tube method was applied and testing functions for sound absorption and insulation performance were designed. The outcomes indicated that in the error test, the error range between the experimental group and the control group was [0.01, 0.18], indicating a high reliability of the experimental results. In the calculation of sound insulation of different specimens at different sound frequencies, when the frequency was 1600Hz, the sound insulation of the control group and experimental group was 65.30dB and 70.14dB, proving the effectiveness of the design method. The above results demonstrate the practicality of integrating BIM technology and impedance tube method in the acoustic performance analysis of wooden structure building walls. This study provides strong technical support for reducing the indoor environment of wooden buildings and improving the comfort of people’s living environment.

## 1. Introduction

With the rapid development of construction technology and increasing attention to building quality, functionality, and sustainability, the construction industry is undergoing unprecedented changes [1]. Building performance refers to various performance parameters and indicators related to buildings, including structure, thermal, acoustic, light environment, safety, water environment performance, and environmental adaptability, etc. The acquisition and analysis of building performance information can be carried out through laboratory testing, simulation calculations, data collection, and other means. Common evaluation tools include building simulation software, testing equipment, sampling, and actual measurement. Building performance information not only has an important impact on building design and construction, but also provides safe, healthy, and comfortable indoor environments for building users.

In recent years, many methods have been proposed by professionals both domestically and internationally to analyze the acoustic performance of building walls. M. Mónica et al. proposed a performance analysis method based on numerical simulation to improve the acoustic performance of stretched membrane roofs. The method combined scalable cross arched ceilings and semi transparent sound-absorbing films for numerical simulation and parameter analysis. The results showed that this method could improve the acoustic performance of high complex volume buildings [2]. L. Yuvaraj et al. investigated the acoustic performance of buried micro-perforated plates by adding manufacturing, experimental impedance tubes, integral method with end correction, and transfer matrix method. The results showed that the introduction of porous materials broadened the sound absorption bandwidth [3]. J. L. Zamora Mestre et al. evaluated the acoustic performance of a lightweight ventilated facade using a double-layer shell structure with a middle non ventilated air chamber, and the results showed that this structure could provide both high air sound insulation effect and controlled ventilation humidity and heat advantages [4]. C. G. Carter et al. conducted measurements from joint characterization to air propagation and impact sound insulation to verify the acoustic performance of the cross laminated timber structure building model [5]. By comparing the measured and predicted acoustic performance values, it was found that the building model had high acoustic performance. E. Roque et al. evaluated the sound insulation performance of lightweight steel structure exterior walls, taking into account the acoustic performance defects of steel structures and the requirements of exterior wall sound insulation performance. They used INSUL software to evaluate the sound insulation performance, and the results showed that appropriate design and material selection can effectively improve the sound insulation performance [6]. H. Kaidouchi et al. established a model using finite element analysis software to study the structural acoustic performance of sandwich panels to obtain the best material for acoustic performance of planar honeycomb sandwich structures. The outcomes indicated that GFRP core materials had better vibration acoustic and sound transmission characteristics compared to FRP surface materials [7]. A. Santoni et al. proposed a prediction model based on the standard EN ISO 12354 to predict the sound attenuation index of building partitions. The model was tested by simulating building partitions made of different materials, and the results showed that the prediction accuracy of the model was high [8]. A. Arjunan et al proposed a method to improve the sound insulation of drywall partitions by using perforated columns to address the sound bridging problem caused by steel columns. The method modelled the acoustic and structural performance through finite element modeling, and the results showed that this method could effectively improve the sound insulation performance of drywall partitions [9]. P. Zhang et al. designed a BIM-based optimization method for the seismic performance of irregular column structures in residential buildings, addressing the issue of poor seismic performance after traditional optimization methods. They utilized BIM technology to construct a building visualization information database model to analyze the parameters of irregular column structures in residential buildings. The results showed that the optimization effect of this method was good [10]. F. Feng et al. designed a building lifecycle assessment method based on BIM technology to evaluate and improve environmental performance at the building level. The method ensured the accuracy of building materials and components through BIM and provided dynamic material updates for design optimization frameworks. The results showed that this method could reduce energy consumption in building design [11]. G. Salvalai et al. used numerical analysis to predict the performance of ventilation facade walls. Two cross laminated wood test boxes equipped with different thermal coating technologies were used to evaluate the surface temperature and indoor air temperature of different wall layers. The results showed that the temperature inside the ventilation facade wall cavity decreased by 8–10°C, indicating good performance [12]. In summary, many scholars have made significant achievements in the field of acoustic performance of building walls. However, although the above research provides multiple evaluation methods for the acoustic performance of building walls, Wooden Structure Buildings (WSBs) are often overlooked in the study of acoustic performance due to their inherent material characteristics and construction methods. As an important component of sustainable building practices, WSBs have good insulation and energy efficiency performance, but their acoustic performance has not been fully evaluated, which limits the potential application of WSBs in terms of acoustic environment comfort.

The rise and application of Building Information Model (BIM) technology has provided new possibilities for the entire process management of building design, construction, and operation. Meanwhile, the Impedance Tube Method (ITM), as an effective acoustic measurement tool, is widely used in the research and evaluation of building acoustic performance. BIM technology provides a detailed description of the physical and functional characteristics of buildings by creating digitized 3D models [13]. This technology can not only improve design efficiency and reduce construction errors, but also provide accurate data basis for building performance evaluation. Through BIM technology, accurate wall geometry and internal structural information can be obtained, providing a basis for the arrangement of impedance tubes. In addition, BIM technology can also simulate and analyze the acoustic environment of buildings, predict and optimize their acoustic performance [14]. The ITM, as a non-destructive detection method, can measure the acoustic impedance (AI) between the wall and the air, thereby evaluating the acoustic performance of building walls. The AI data obtained through the ITM can further calculate the acoustic performance parameters of the wall, such as transmission loss, sound absorption coefficient (SAC), etc. However, although the above studies provide multiple evaluation methods for the acoustic performance of building walls, WSBs are often overlooked in the study of acoustic performance due to their inherent material characteristics and construction methods. At present, WSBs are still in the development stage, and as an important component of sustainable building practices, they have good insulation and energy-saving performance. However, their acoustic performance has not been fully evaluated, which limits the potential application of WSBs in the comfort of acoustic environments. Therefore, there is an urgent need for an acoustic performance evaluation technology for WSBs. By accurately analyzing the acoustic performance of WSBs, technical support can be provided for the noise reduction work of WSBs, and a more comfortable living environment can be provided for residents.

In view of this, BIM technology and ITM are integrated in this study. First, a building performance test system based on BIM technology is constructed, using Revit, Rhino Inside Revit, Grasshopper, and Ladybug Tools as the main technical tools, a Revit model was developed as the basic model for building performance simulation, and the simulation data was integrated into the Revit model. Then, based on the ITM, the sound absorption performance was tested using the transfer function dual microphone method, and the sound insulation performance was tested using the transfer function four microphone method. The designed sound absorption performance and sound insulation performance functions were applied to the performance testing system of WSBs to achieve accurate prediction of the acoustic performance of WSBs.

The innovation of the research lies in combining BIM technology with ITM to evaluate the acoustic performance of WSBs. Performance data of WSBs are obtained through performance integrated BIM, and ITM is used to analyze the acoustic performance of WSBs, effectively verifying the sound absorption and insulation performance of WSB under different conditions.

The contribution of the research is that the design method can provide theoretical basis and experimental support for the structural optimization of the wooden structure wall, promote the engineering application of the wooden structure building, and provide a new idea for the acoustic performance analysis of other special structure building walls.

The research content includes five parts. The first part is the background introduction of building acoustic performance analysis, BIM technology, and ITM. The second part is the current research status of BIM technology, ITM, and acoustic performance both domestically and internationally. The first section of the third part is the design of a wood structure building performance testing system based on BIM technology, and the second section is the sound absorption performance testing function designed based on the ITM. The fourth part is the acoustic performance analysis of wooden structure walls by integrating BIM technology and ITM. The fifth part is a summary of the entire article and proposes the shortcomings of the research.

## 2. Acoustic performance of wood structure building walls based on BIM technology and ITM

The research integrates BIM technology and ITM to comprehensively evaluate the acoustic performance of WSBs. Firstly, a performance testing system for WSBs based on BIM technology is constructed to simulate and analyze acoustic behaviors. Then, a testing function for measuring sound absorption performance is designed using the ITM to quantitatively test the SAC of materials. Finally, testing functions for evaluating the sound insulation performance of wall structures are designed to assess their soundproofing efficiency.

### 2.1 Design of a performance testing system for wooden structure buildings based on BIM technology

**Fig 1:**
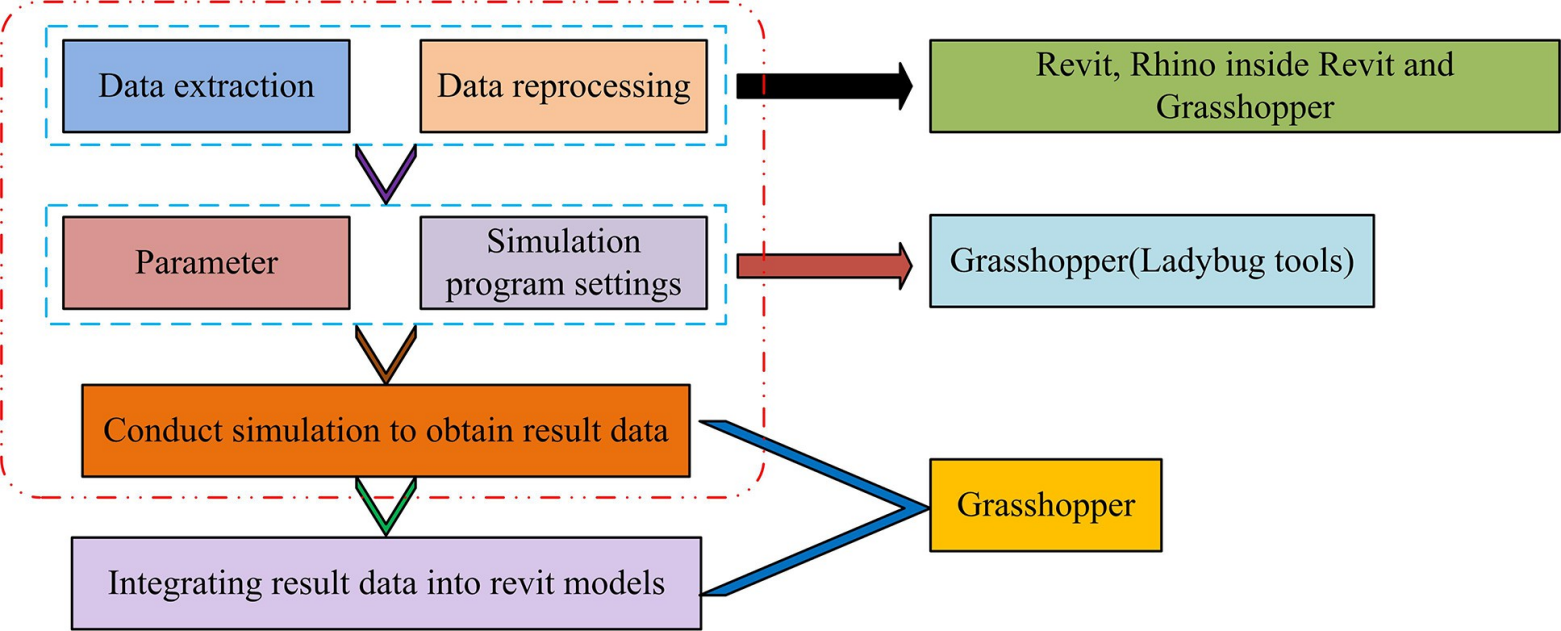
P-BIM technical framework.

BIM, as a commonly used technology for integrating and processing building information, can integrate relevant parameters such as geometric shape, structure, materials, and systems of buildings. BIM supports multidisciplinary collaboration and plays an important role in the construction and management of modern architecture. Meanwhile, BIM contains rich information that can provide data support for building performance simulation. Through BIM, designers can simulate the propagation path and energy loss of sound waves in buildings, thereby helping designers determine the acoustic measures and appropriate materials to be taken to minimize noise interference to the greatest extent possible [15, 16]. However, the information integrated by BIM is the material composition of the building body, without the performance information of the building. Therefore, the study integrates building performance simulation data into BIM to build a Performance Integrated Building Information Model (P-BIM). This system is used to simulate the acoustic properties of wooden buildings to analyze their acoustic properties qualitatively and quantitatively with the specific framework shown in Fig 1.

**Fig 2:**
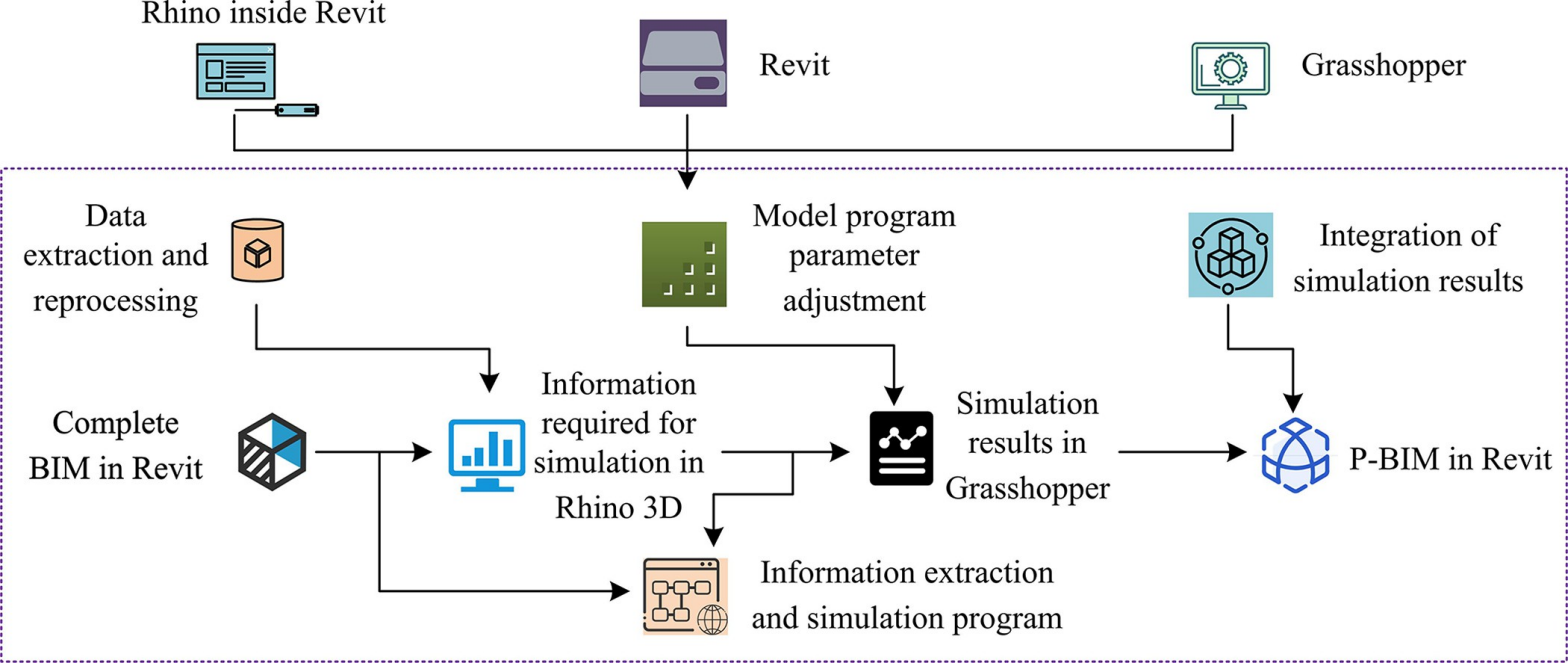
The flow of data information in BIM integration technology.

In Fig 1, P-BIM can be divided into the generation of simulation data and the input and storage of simulation data. Data generation includes the extraction and reprocessing of model data, simulation program setting and parameter adjustment, simulation and obtaining simulation result data. The input and storage of simulation data integrate the simulation result data into the BIM model. The technical tools used for extracting and reprocessing model data include Revit, Rhino inside Revit, and Grasshopper. Among them, Revit is a BIM software that can be used to extract building model data. Rhino inside Revit is a plugin used to use Rhino and Grasshopper in Revit, which can make model data extraction more efficient. Grasshopper is a visual programming tool typically used in conjunction with Rhino to reprocess model data. The technical tool used for setting and parameter adjustment of simulation programs is Grasshopper (Ladybug tools), which is a plugin of Grasshopper specifically used for building environment analysis and simulation. By adjusting the parameters of the Ladybug tool, the simulation program can be set and adjusted to obtain accurate simulation results. The technical tool used for simulation and data integration is Grasshopper, which can integrate simulation results data into the BIM, enabling the BIM to include building performance simulation data, thereby more comprehensively evaluating various performance parameters of the building. In the construction of P-BIM, the flow of data information in BIM integration technology is shown in Fig 2.

In Fig 2, after the complete BIM design in Revit, the designed model needs to be imported into Rhino3d for further simulation and analysis. Rhino3d is a powerful 3D modeling software that can perform advanced geometric and morphological transformations on building models. By simulating the required information in Rhino3d, the designed scheme can be further optimized and improved. Next, it uses the Grasshopper plugin in Rhino3d to simulate the design results. Grasshopper is a visual programming tool for parametric design and modeling, which can create complex algorithms and programs through visualization. In Grasshopper, the simulation process of design can be constructed by connecting various components, including structural analysis, solar exposure analysis, energy simulation, etc. After completing the simulation and analysis in Rhino3d and Grasshopper, it will import the results into Revit again to improve the BIM model. By integrating simulation results from Rhino3d and Grasshopper with BIM models in Revit, more comprehensive and accurate design solutions can be obtained, improving design efficiency and quality. Finally, the required information segments and simulation program segments are extracted through the general integration program provided by Grasshopper. These information segments and simulation program segments can be used in subsequent design processes, such as further optimization, analysis, and evaluation. After integrating building performance into the P-BIM model, relevant data processing and performance analysis of building structures can be carried out.

### 2.2 Design of sound absorption performance test function based on ITM

**Fig 3:**
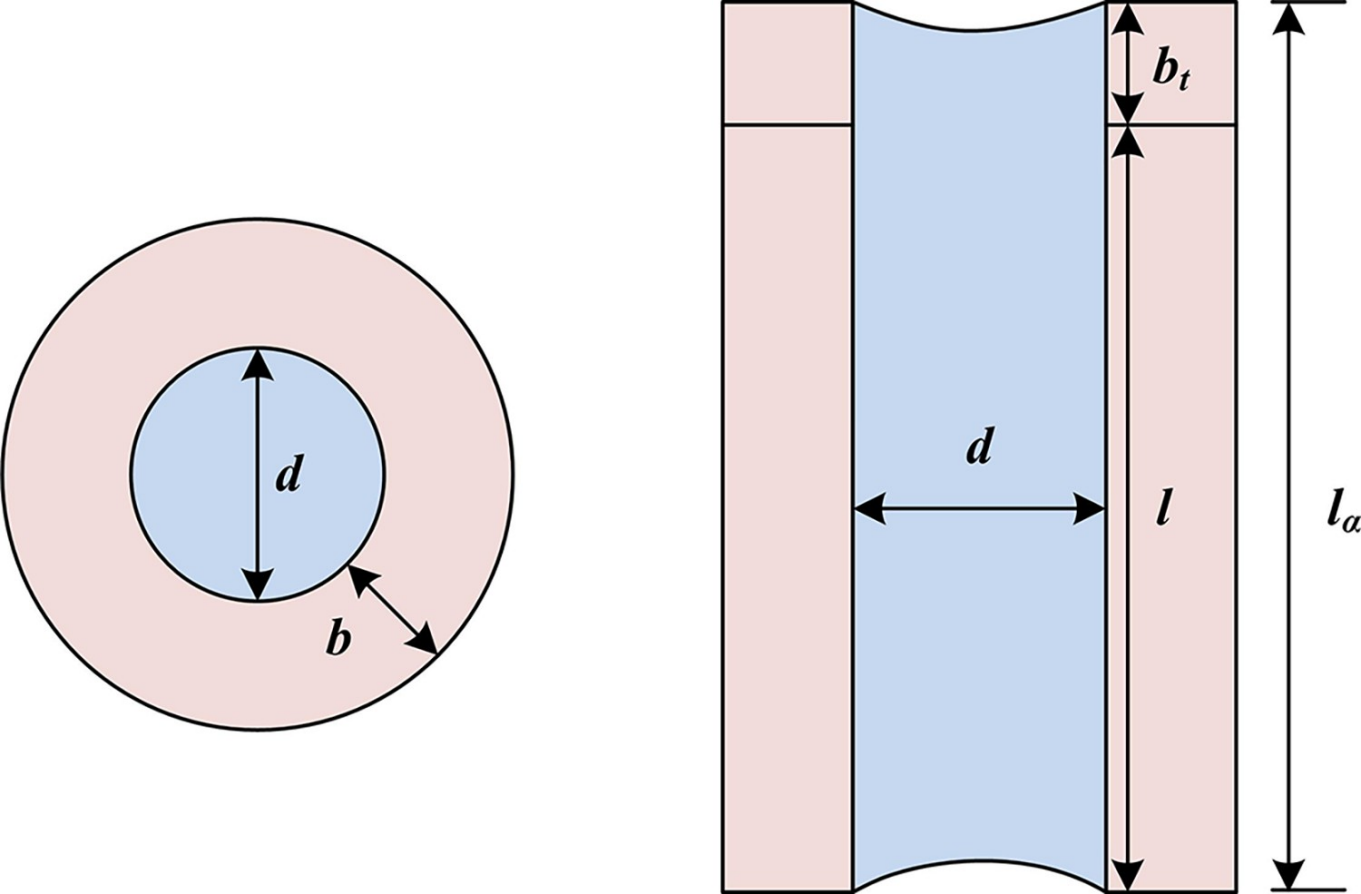
Cross and longitudinal section structure embedded in aperture.

The acoustic materials and structures used in architectural acoustics research and noise control engineering have two aspects of acoustic performance: sound absorption and insulation performance. Therefore, the acoustic performance testing of acoustic materials and acoustic structures includes two parts. The first is the sound absorption performance testing, mainly the measurement of the SAC parameters. The second is sound insulation performance testing, mainly testing the sound insulation parameters [17, 18]. The research mainly focuses on the acoustic effectiveness analysis of WSB walls. In traditional research methods, ITM is the standard means to evaluate the sound absorption performance of materials, which evaluates their acoustic characteristics by measuring their AI and SAC. With the widespread application of BIM technology in the construction industry, its integration with acoustic testing methods presents new research directions and potential value. BIM is a multidimensional information model based on digital technology that can be updated in real-time, providing comprehensive information support for the design, construction, management, and maintenance of buildings. In acoustic performance analysis, integrating ITM with BIM can achieve more accurate prediction and simulation of the sound absorption effect of building materials. This integration allows researchers to quickly adjust and optimize the acoustic properties of materials in a virtual environment, thereby predicting the acoustic performance of the entire building structure. Therefore, the study conducted sound absorption performance testing on wall unit structures using ITM in a wood structure building performance testing system based on BIM technology, and embeds holes and slots in the cavity of the wall unit structure to enhance its sound absorption ability. The cross-sectional structure of the embedded aperture is expressed in Fig 3.

In Fig 3, when sound waves enter the cavity resonant sound-absorbing structure, they will reflect back and forth between the holes, leading to the absorption of sound energy inside the structure. To achieve perfect sound absorption, it is necessary to adjust the absorption structure or the impedance between the absorption material and the air, which is generated when sound waves propagate inside the absorption material [19]. During the propagation of sound waves, the interaction between them and the medium generates viscous forces, and there is also heat transfer between the two, resulting in viscous and thermal losses. The parameter relationship is shown in Eq (1).


$$\left\{ \begin{array}{l} K_{\nu }^{2}=-\frac{Jw\rho_{0}}{\mu }\\ K_{h}^{2}=-\frac{Jw\rho_{0}C_{p}}{\mu } \end{array}\right.$$ (1)

In Eq (1), *K*_*v*_ means the parameter viscous wave number of viscous loss; *K*_*h*_ denotes the parameter thermal wave number of thermal loss; *J* represents the first type first-order Bessel function; *w* represents angular frequency; *C*_*p*_ represents constant pressure specific heat; *μ* represents the dynamic viscosity of air, in *Pa*⋅*s*. The functional expressions of the viscous and thermal fields can be calculated from the viscous and thermal wave numbers, as shown in Eq (2).


$$\left\{ \begin{array}{l} \varphi_{h}=\frac{J_{2}(K_{h}d/2)}{J_{0}(K_{h}d/2)}\\ \varphi_{\nu }=\frac{J_{2}(K_{v}d/2)}{J_{0}(K_{v}d/2)} \end{array}\right.$$ (2)

In Eq (2), *φ*_*h*_ indicates the function of the thermal field; *φ*_*v*_ represents the function of the viscous field; *J*_2_ represents the first and second order Bessel functions; *d* represents the diameter; *J*_0_ represents the first and zero order Bessel functions. The next step is to calculate the complex air density and complex wave number, as shown in Eq (3).


$$\left\{ \begin{array}{l} \rho_{c}=\frac{\rho_{0}}{\varphi_{\nu }}\\ K_{c}^{2}=K_{0}^{2}(\frac{r-\varphi_{h}(r-1)}{\varphi_{\nu }}) \end{array}\right.$$ (3)

In Eq (3), *ρ*_*c*_ represents the complex air density,; *K*_*c*_ represents the complex wave number; *r* represents the adiabatic index of the air. In walls, there is usually a backing cavity, which is a cavity structure located inside the wall to raise the sound reflection and absorption characteristics of the wall. Therefore, it needs to calculate the AI of the backing cavity, as shown in Eq (4).


$$Z_{\nu }=-\frac{JS\rho_{c}c_{c}}{wV}$$ (4)

In Eq (4), *Z*_*v*_ represents the corrected AI of the backing cavity; *S* represents the cross-sectional area embedded in the hole, and *J* represents the complex sound velocity. The next step is to embed the AI of the aperture, and the calculation method is indicated in Eq (5).


$$\left\{ \begin{array}{l} Z_{0}=-\frac{J\rho_{0}wl_{a}}{wV}\\ V=A_{c}L-S^{\prime }l \end{array}\right.$$ (5)

In Eq (5), *Z*_0_ represents the AI of the embedded aperture; *l*_*a*_ represents the total longitudinal length of the embedded aperture; *V* represents the volume of the irregular part inside the wall structure cavity; *A*_*c*_ refers to the cross-sectional area inside the wall unit; *A*_*c*_ represents the length inside the wall unit; *S*′ means the cross-sectional area of the embedded aperture; *l* represents the length embedded into the wall unit. The AI of the entire wall structure can be calculated by using the AI of the backing cavity and the embedded aperture, as shown in Eq (6).


$$Z=-\frac{J\rho_{0}wl_{a}}{wV}-\frac{JS\rho_{c}c_{c}}{wV}$$ (6)

**Fig 4:**
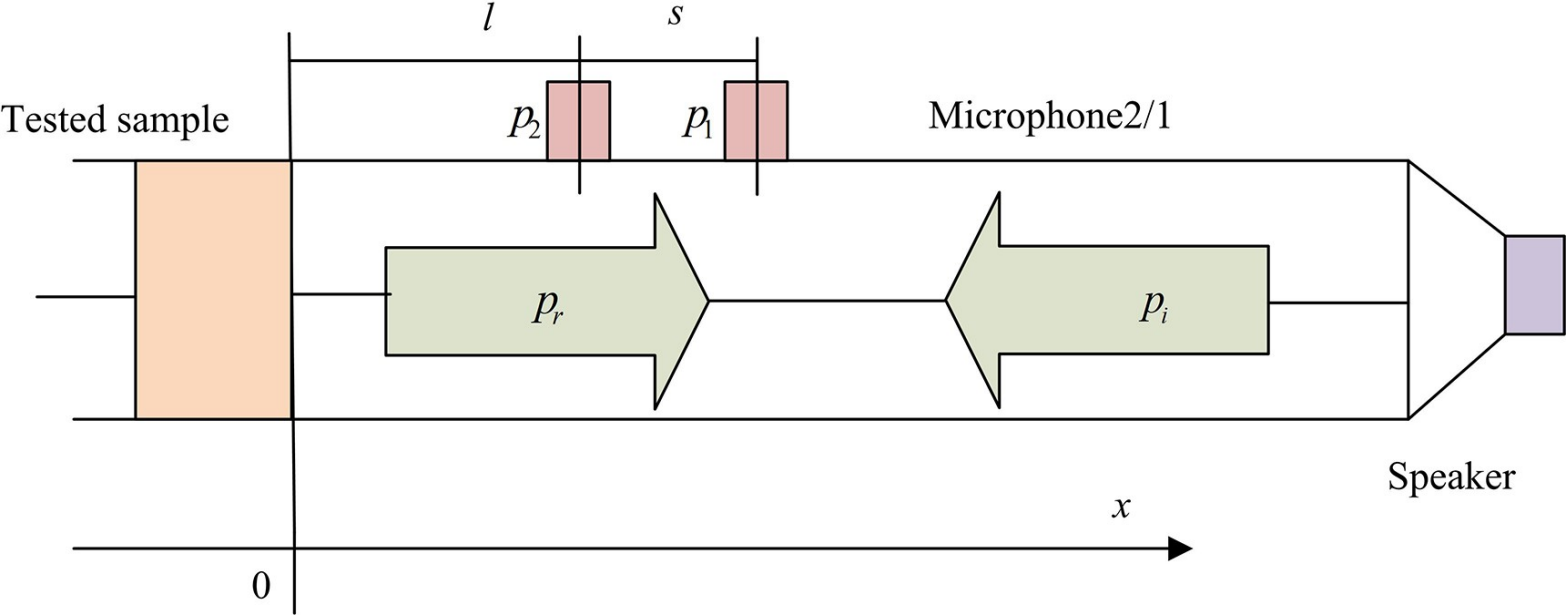
Plan view of sound absorption performance testing using dual microphone method.

In Eq (6), *Z* represents the AI of the entire wall unit structure. The method used for testing the sound absorption effectiveness of impedance tubes is the transfer function dual microphone method, and its planar structure is shown in Fig 4.

The transfer function dual microphone method is a method of measuring sound transmission characteristics. Two microphones are adopted, one as the signal source and the other as the receiver. By comparing the differences in signals received by the signal source and receiver, the frequency response of the transfer function can be calculated, describing the characteristics of attenuation and distortion during sound transmission. The test sample is loaded into one end of the impedance tube, and the plane wave in the tube is generated by the sound source. The sound pressure at two positions near the test sample is measured to obtain the sound transfer functions of the two sensor signals, and then the normal incident SAC of the test sample is calculated. The calculation method for reflected and incident sound waves during performance testing is shown in Eq (7).


$$\left\{ \begin{array}{l} I=P_{I}e^{JK_{c}x}\\ R=P_{R}e^{-JK_{c}x} \end{array}\right.$$ (7)

In Eq (7), *I* severs as the incident sound wave; *R* stands for the reflected sound wave; *P*_*I*_ and *P*_*R*_ represent the amplitude of the measured sound pressure on the impedance tube reference plane; *e* represents the natural constant; *x* represents the testing distance. The sound pressure at the positions of the two microphones can be calculated based on the reflected and incident sound waves. The calculation method is shown in Eq (8).


$$\left\{ \begin{array}{l} P_{1}=P_{I}e^{JK_{c}(s+l)}+P_{R}e^{-JK_{c}(s+l)}\\ P_{2}=P_{I}e^{JK_{c}l}+P_{R}e^{-JK_{c}l} \end{array}\right.$$ (8)

In Eq (8), *P*_1_ and *P*_2_ represent the sound pressure at the two microphones; *s* represents the distance between them; *l* represents the distance between the test and the nearest microphones. The transfer function expressions for the incident and reflected sound waves at the two microphones are shown in Eq (9).


$$\left\{ \begin{array}{l} H_{I}=\frac{P_{2I}}{P_{1I}}=e^{-JK_{c}s}\\ H_{R}=\frac{P_{2R}}{P_{1R}}=e^{JK_{c}s} \end{array}\right.$$ (9)

In Eq (9), *H*_*I*_ represents the transfer function of the incident sound wave; *H*_*R*_ represents the transfer function of the reflected sound wave; *P*_1*I*_ and *P*_2*I*_ represent the incident sound pressure at the two microphones; *P*_1*R*_ and *P*_2*R*_ represent the reflected sound pressure at the two microphones. According to Eq (9), the transfer function of the total sound field can be calculated, as shown in Eq (10).


$$\left\{ \begin{array}{l} H=\frac{P_{2}}{P_{1}}=\frac{e^{JK_{c}l}+re^{-JK_{c}l}}{e^{JK_{c}(s+l)}+re^{-JK_{c}(s+l)}}\\ r=\frac{H-H_{I}}{H_{R}-H}e^{2JK_{c}(s+l)} \end{array}\right.$$ (10)

In Eq (10), *H* and *r* represent the transfer function of the total sound field and the reflection coefficient, respectively. Finally, the SAC and normal AI of the test sample can be obtained, as shown in Eq (11).


$$\left\{ \begin{array}{l} \alpha =1-{\left|r\right|}^{2}\\ Z=\frac{1+r}{1-r}\rho c_{0} \end{array}\right.$$ (11)

**Fig 5:**
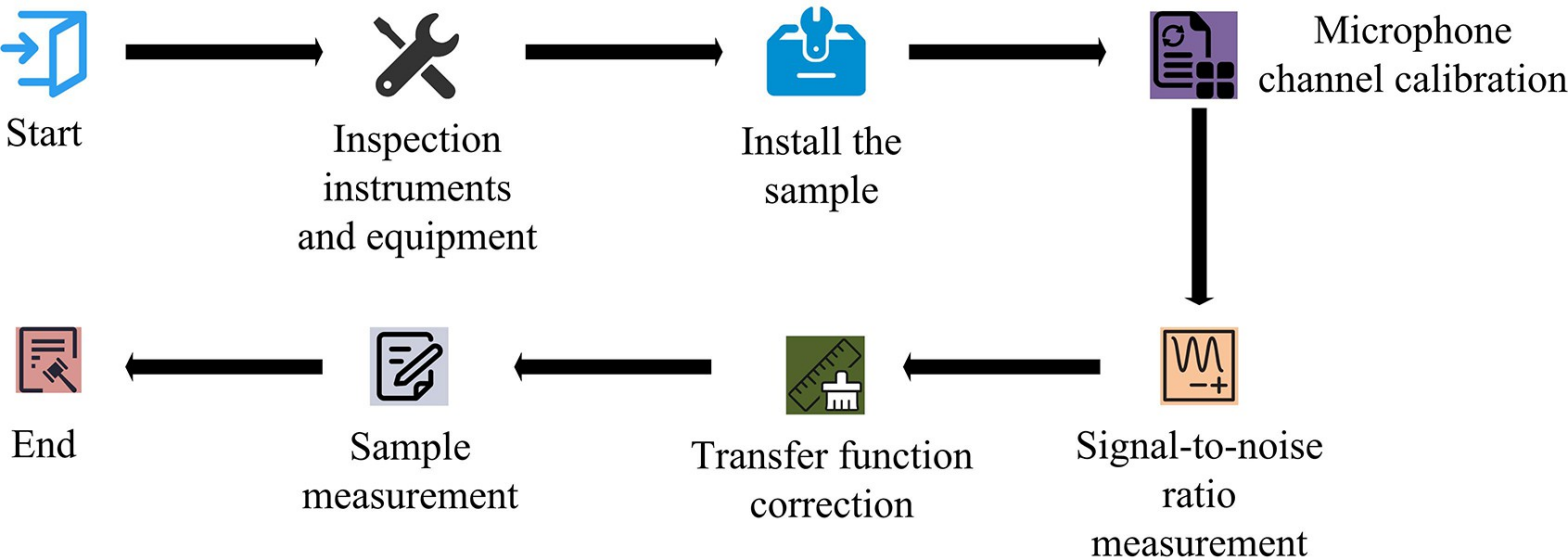
Test steps for sound absorption performance of ITM.

In Eq (11), *α* represents the SAC; *Z* represents the normal AI; *ρ* represents the density of the medium, in units of *kg*/*m*^3^; *c*_0_ represents the propagation speed of sound waves, in units of *m*/*s*. The steps for conducting sound absorption performance testing using the ITM are shown in Fig 5.

In Fig 5, first, it needs to check and confirm the normal working status of the testing instruments and equipment, and then a calibrator is used to calibrate the sound channel of the microphone to ensure that the frequency response and level response of the microphone meet the standards. The next step is to generate a reference signal without stray interference using a signal generator, and measure the output signal of the microphone without a sample to determine the signal-to-noise ratio of the signal generator. Next, it will measure the output signal of the microphone with a sample, and correct the transfer function by comparing it with the signal without a sample to eliminate the influence of the sample on the measurement outcomes. Finally, with correcting the transfer function, the sound absorption effectiveness of the sample to sound waves is measured.

### 2.3 Design of sound insulation performance test function based on ITM

**Fig 6:**
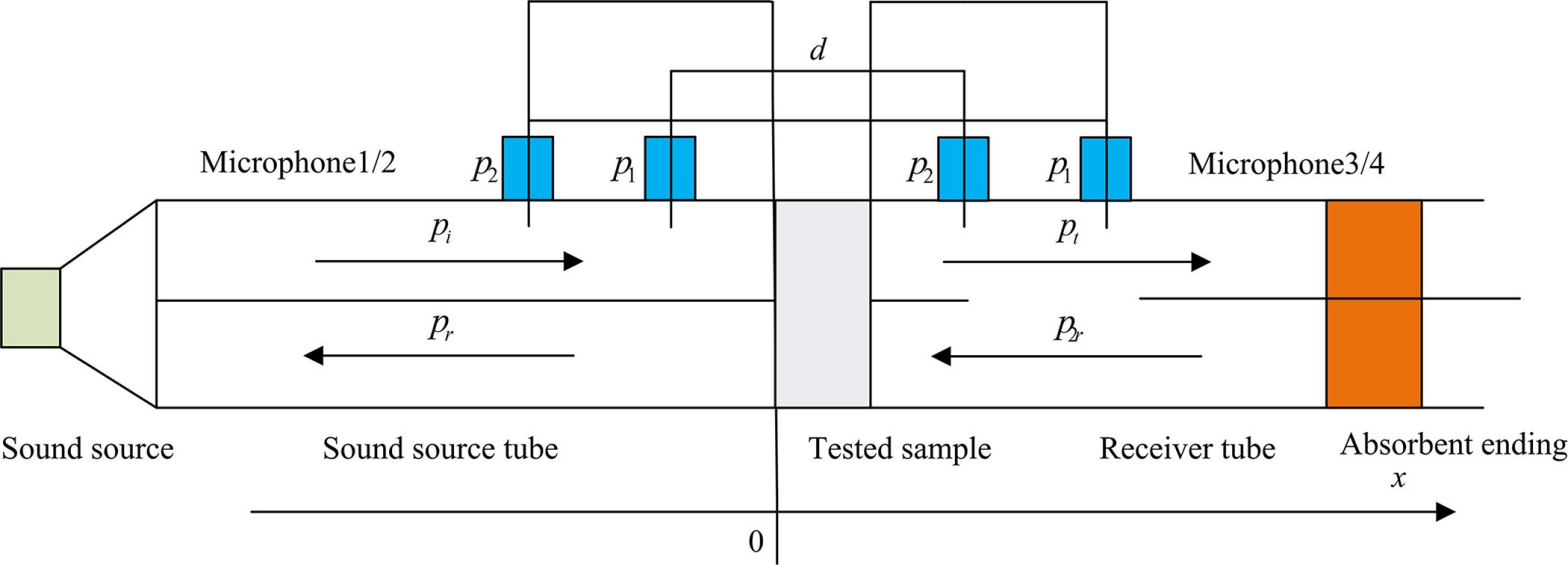
Plan view of sound insulation performance testing using the four microphone method.

Sound insulation performance refers to the ability of materials, components, or structures to block the transmission of sound waves, and its measurement is based on the sound insulation index [20]. The testing method used in the study is the transfer function four transmission method, and the plane diagram during the testing is shown in Fig 6.

In Fig 6, the transfer function four microphone method is a commonly used experimental method based on acoustic transmission theory, which calculates the sound insulation index of materials or structures by measuring sound pressure levels and transfer functions. The transfer function four microphone method uses four microphones placed on the sound source side, on both sides of the tested sample, and on the sound absorbing end side. The source side microphone emits sound signals, the microphones on both sides of the tested sample are used to measure the sound pressure level before and after the obstacle, and the sound absorbing end microphone is used to measure the sound pressure level on the target side. From these measured values, the transfer function, which is the ratio of the sound pressure levels before and after the obstacle, can be calculated. By using the transfer function, the sound insulation index can be calculated. The sound insulation index is an indicator utilized to assess the sound insulation of a material or structure, indicating its ability to isolate sound waves [21]. The calculation of sound insulation index is based on acoustic transmission theory and the difference in sound pressure levels before and after obstacles. It sets the sound pressure at the four microphones from left to right as *P*_3_, *P*_4_, *P*_5_, and *P*_6_, and the calculation is denoted in Eq (12).


$$\left\{ \begin{array}{l} P_{1}=P_{I}e^{JK_{c}x_{1}}+P_{R}e^{-JK_{c}x_{1}}\\ P_{2}=P_{I}e^{JK_{c}x_{2}}+P_{R}e^{-JK_{c}x_{2}}\\ P_{3}=P_{T}e^{JK_{c}x_{3}}+P_{2R}e^{-JK_{c}x_{3}}\\ P_{4}=P_{T}e^{JK_{c}x_{4}}+P_{2R}e^{-JK_{c}x_{4}} \end{array}\right.$$ (12)

In Eq (12), *x*_1_, *x*_2_, *x*_2_, and *x*_4_ respectively represent the distance between the four microphones and the tested sample; *P*_*T*_ expresses the plane transmitted sound wave generated by the incident wave *P*_*I*_ passing through the tested sample; *P*_2*R*_ represents the plane reflected wave generated by *P*_*T*_ passing through the absorbing end. The next step is to calculate the inlet and outlet, reflected, plane transmission, and reflected sound pressure at the absorption end, as shown in Eq (13).


$$\left\{ \begin{array}{l} P_{I}=\frac{P_{1}-P_{2}e^{-JK_{c}(x_{1}-x_{2})}}{e^{JK_{c}(x_{1}-x_{2})}-e^{-JK_{c}(x_{1}-x_{2})}}e^{-jkx_{2}}\\ P_{R}=\frac{P_{1}-P_{2}e^{JK(x_{1}-x_{2})}}{e^{-JK_{c}(x_{1}-x_{2})}-e^{JK_{c}(x_{1}-x_{2})}}e^{jkx_{2}}\\ P_{T}=\frac{P_{3}e^{JK(x_{4}-x_{3})}-P_{4}}{e^{JK_{c}(x_{4}-x_{3})}-e^{-JK_{c}(x_{4}-x_{3})}}e^{jkx_{3}}\\ P_{2R}=\frac{P_{3}e^{-JK(x_{4}-x_{3})}-P_{4}}{e^{-JK_{c}(x_{4}-x_{3})}-e^{JK_{c}(x_{4}-x_{3})}}e^{-jkx_{3}} \end{array}\right.$$ (13)

In Eq (13), the incident and reflected sound pressure are calculated from the sound pressure *P*_1_ and *P*_2_ at the microphone, while the plane transmission sound pressure and the sound pressure at the absorption end are calculated from the sound pressure *P*_3_ and *P*_4_ at the microphone. Then, the sound pressure transmission coefficient of the test sample can be calculated, as shown in Eq (14).


$$\tau =\frac{\sin(K_{c}x_{1}-K_{c}x_{2})P_{3}e^{JK_{c}(x_{4}-x_{3})}-P_{4}}{\sin(K_{c}x_{4}-K_{c}x_{3})P_{1}-P_{2}e^{-JK_{c}(x_{1}-x_{2})}}e^{JK_{c}(x_{2}+x_{3})}$$ (14)

In Eq (14), *τ* expresses the sound pressure transmission coefficient of the test sample. Finally, the sound insulation amount is calculated using the sound pressure transmission coefficient, as shown in Eq (15).


TL=−20lg|τ| (15)

**Fig 7:**
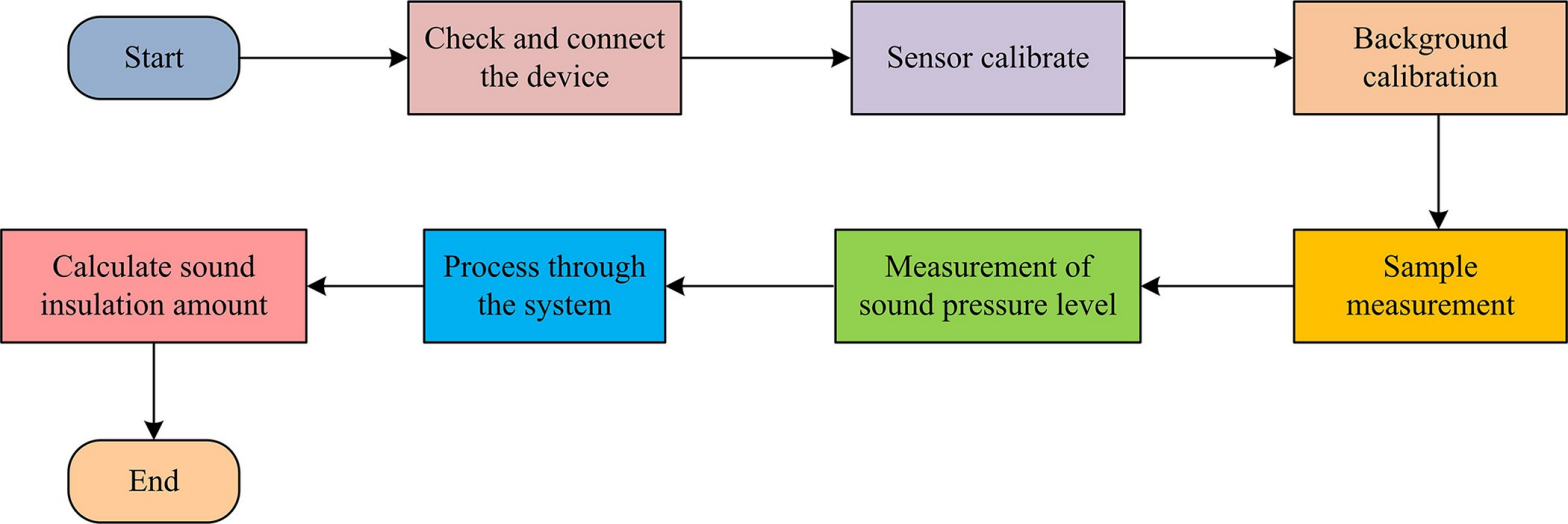
The process of ITM for sound insulation performance testing.

In Eq (15), *TL* represents the sound insulation amount, and lg represents a logarithmic function with a base of 10. The process of conducting sound insulation performance testing using ITM is shown in Fig 7.

In Fig 7, first is to set the inner diameter and testing frequency of the impedance tube, select the appropriate inner diameter based on the characteristics of the sample, and set the testing frequency range. Then it performs sensor calibration to ensure the accuracy and consistency of the sensors used in the test. Typically, a reference object with known AI is used to calibrate the sensor. Before testing, it is necessary to measure and correct background noise to eliminate the impact of environmental noise on the test results. Next, it places the sample at the inlet and outlet of the impedance tube and measures its sound pressure level difference. During this process, multiple measurements of different sound incidence angles are required at each testing frequency to obtain the average value. Finally, the data obtained from the test will be further processed and statistically analyzed by constructing a weighted matrix with different indicator weights, and the sound insulation of the sample will be calculated using a formula. BIM technology can efficiently obtain information about building walls, while ITM can evaluate the acoustic performance of building materials by measuring their AI. Therefore, by integrating BIM technology with ITM, BIM technology is used to integrate and manage various data obtained, including acoustic performance data tested by ITM, to facilitate analysis and ensure the accuracy and efficiency of sound absorption performance testing. This integrated technology can avoid the trial and error costs in traditional methods, while also simulating complex architectural acoustic environments. Compared with traditional acoustic evaluation methods, it can achieve rapid evaluation and optimization of acoustic performance through integrated methods.

## 3.Analysis of acoustic performance results of wooden building walls based on BIM technology and ITM

This section mainly verifies the sound absorption and insulation performance of BIM technology and ITM integrated method in building walls from the aspects of material thickness, structure, and different acoustic frequencies.

### 3.1Analysis of wall acoustic absorption effect based on BIM technology and ITM

**Fig 8:**
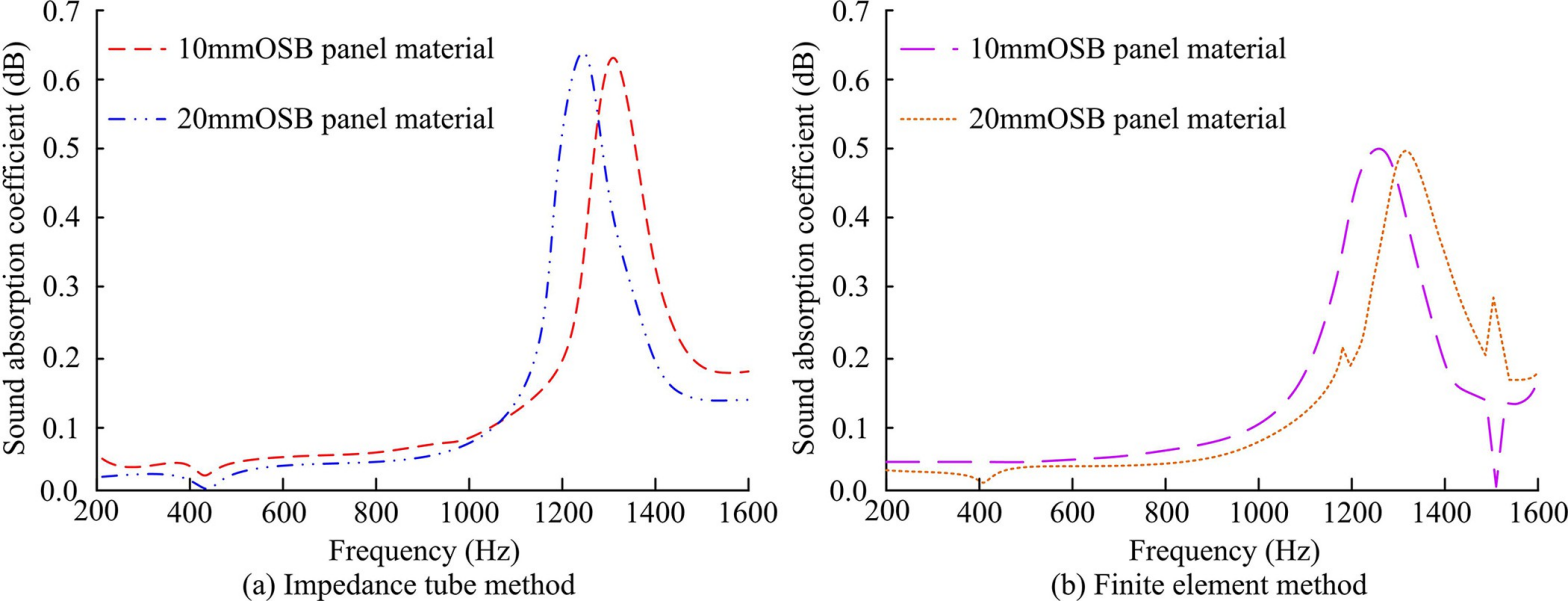
The relationship between SAC and frequency of OSB panel materials with different thicknesses.

To achieve acoustic performance analysis in BIM environment, the absorption coefficient and AI data obtained by ITM were input into the BIM model. The model could simulate the propagation and reflection of sound waves in wall structures based on these parameters, thereby predicting the sound absorption effect under different building materials and structural designs. The study selected an impedance tube with a model of B&K4206 and a large inner diameter of 100mm. The distance between the microphones was set to 50mm, the distance between the microphone and the surface of the test sample was set to 150mm, and the test frequency was set between 90-1600Hz. In addition, two 4187 dual microphones, one power amplifier, and one sound calibrator were selected as supporting facilities. By designing a performance testing system based on BIM technology for sound absorption performance analysis, the relationship curves between SAC and frequency were calculated for OSB panel materials of different thicknesses. The OSB panel materials with thicknesses of 10mm and 20mm were taken and compared with the wall sound absorption performance results based on finite element method. It intended to evaluate the relationship between the sound absorption capacity of wall units and material thickness. The improvement in sound absorption performance of wall units were compared and analyzed after adding hole and groove structures and filling sound-absorbing cotton as acoustic optimization measures. The finite element method is a powerful numerical simulation tool that can provide detailed information about complex systems. By comparing the results of wall sound absorption performance based on finite element method, the accuracy and reliability of the SAC obtained by ITM could be verified. The comparison results are shown in Fig 8.

**Fig 9:**
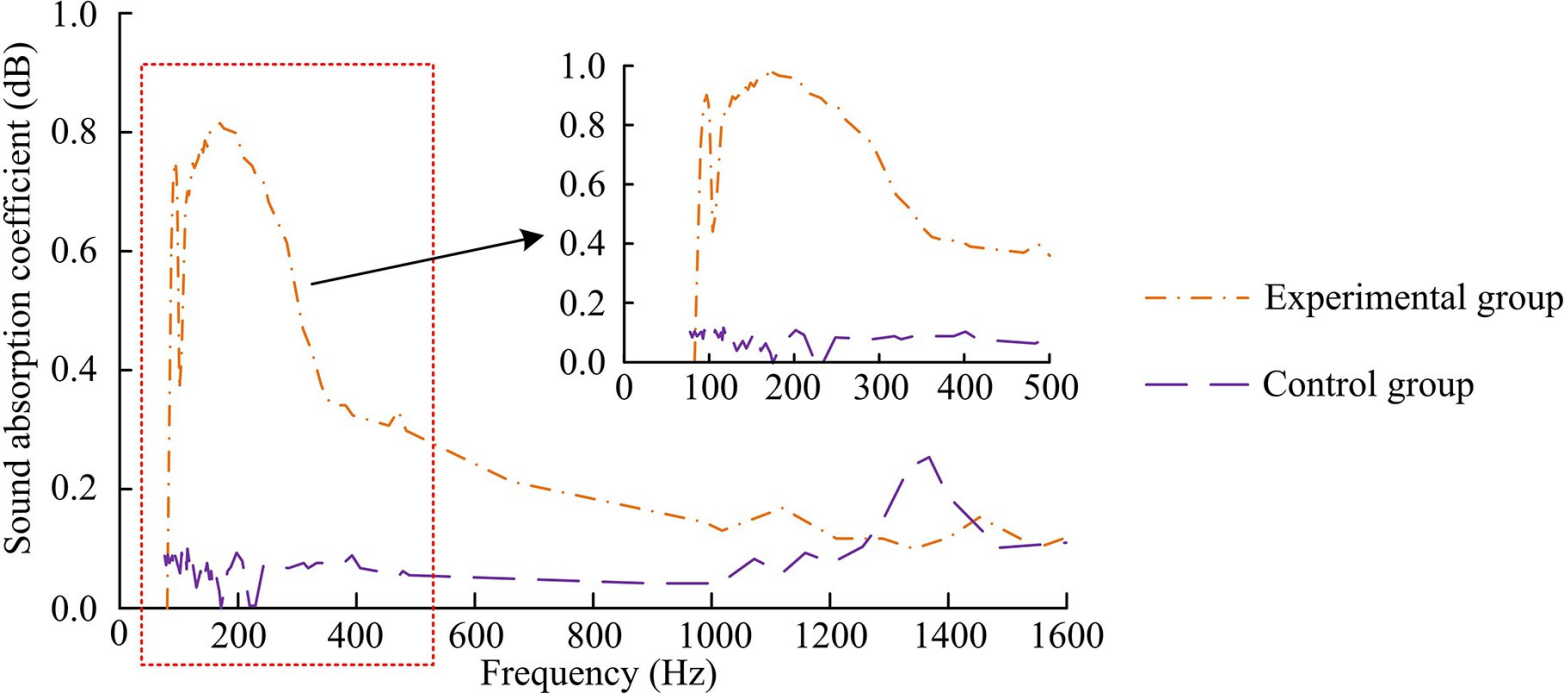
Changes in SAC of two sets of specimens under different acoustic frequencies.

Fig 8(A) and 8(B) show the variation of sound absorption performance based on designed method and finite element method with frequency under different thicknesses of OSB panel materials, respectively. From Fig 8(A), when the thickness of the OSB panel material was 10mm, the SAC was 0.051 at a frequency of 400Hz, 0.083 at a frequency of 800, and 0.182 at a frequency of 1600Hz. When the thickness of the OSB panel material was 20mm, the SAC was 0.042 at a frequency of 250Hz, 0.069 at a frequency of 800, and 0.147 at a frequency of 1600Hz. From Fig 8(B), when the thickness of the OSB panel material was 10mm, the SAC was 0.042 at a frequency of 400Hz, 0.074 at a frequency of 800, and 0.161 at a frequency of 1600Hz. When the thickness of the OSB panel material was 20mm, the SAC was 0.037 at a frequency of 250Hz, 0.043 at a frequency of 800, and 0.179 at a frequency of 1600Hz. The trend of the SAC changing with frequency was consistent with that based on the ITM, but its SAC would fluctuate with increasing frequency. The above results proved that the method designed in the study had a good sound absorption effect. The next step was to select two different sets of wall units and divide them into an experimental group and a control group. The former consisted of wall unit structures embedded in holes and filled with sound-absorbing cotton OSB panel material inside, while the latter consisted of wall unit structures filled with the same material inside, with three each type of structure. The variation curves of the SAC of two sets of specimens under different sound wave frequencies were calculated, as shown in Fig 9.

From Fig 9, when the sound frequency was 100Hz, the SAC of the control group and experimental group was 0.08 and 0.50, respectively. This result indicated that within the low-frequency range, the sound absorption of the experimental group was significantly better than that of the control group. When the sound wave frequency increased to 110Hz, the SAC of the control group decreased to 0.07, while the SAC of the experimental group increased to 0.68. At 185Hz, the SAC of the control group further decreased to 0.01, while the SAC of the experimental group reached 0.81. The above results indicated that as the frequency of sound waves increased, the sound absorption performance of the experimental group gradually surpassed that of the control group. When the sound wave frequency was 1365Hz, the SAC of the control group was 0.25, while the SAC of the experimental group decreased to 0.11. This result indicated that in the high-frequency range, the sound absorption performance of the control group was better than that of the experimental group, indicating that in the high-frequency range, the pore groove structure did not bring the expected improvement in sound absorption performance. In summary, the experimental data supports that the embedded hole groove wall unit has better sound absorption ability than traditional walls in the mid to low frequency range, while its performance in the high frequency range needs further optimization. These findings demonstrate the effectiveness of analyzing the sound absorption performance of walls through design methods and have important value in guiding the acoustic design of actual building walls. The next step was to conduct error analysis on the two groups, and the outcomes are indicated in Table 1.

**Table 1:** Error analysis of the experimental and control groups.

Frequency (Hz)	Experimental group	Control group	Error
200	0.80	0.62	0.18
400	0.34	0.49	0.15
600	0.23	0.40	0.17
800	0.18	0.28	0.10
1000	0.14	0.20	0.06
1200	0.12	0.11	0.01
1400	0.12	0.08	0.04
1600	0.12	0.05	0.07

**Fig 10:**
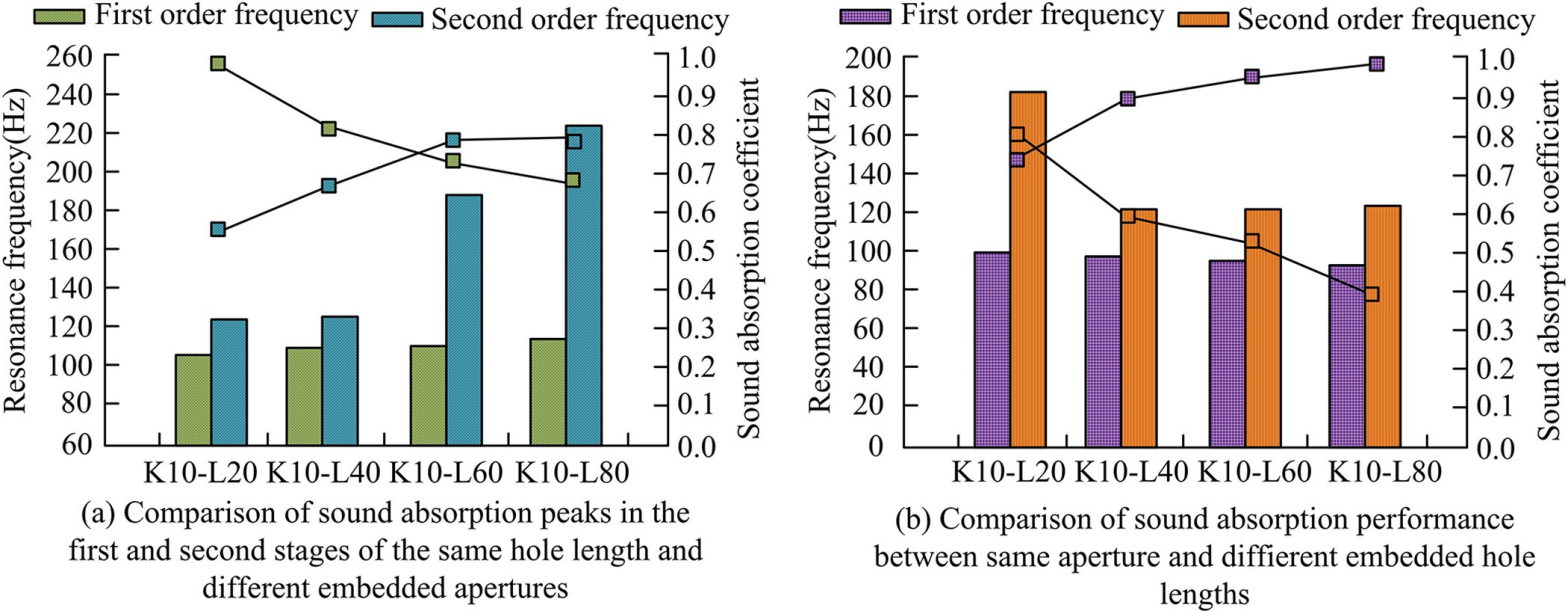
Resonance frequency and absorption peak under different conditions.

From Table 1, the error range between the two groups was between 0.01 and 0.18. When the sound absorption frequency was 200Hz, the value of the experimental group and the control group was 0.80 and 0.62, respectively, with an error value of 0.18, indicating that there was a significant difference in numerical values between the two groups under low-frequency conditions. As the sound absorption frequency increased, the values of the two groups gradually approached, and the error value also gradually decreased. At a sound absorption frequency of 1200Hz, the minimum error value between the two groups was 0.01, indicating that the numerical difference between the two groups was small at high frequencies. Overall, the average error was 0.097, indicating a small difference between the two groups, and the reliability of the experimental findings was high. The above conclusion can be drawn that the SAC of the experimental group is higher than that of the control group. Finally, the first and second order sound absorption peaks were compared under the same hole length, different embedded apertures, and the same embedded aperture with different hole lengths. The results are shown in Fig 10.

Fig 10(A) shows the resonance frequency and absorption peak under the same hole length but different embedding apertures, while Fig 10(B) shows the resonance frequency and absorption peak under the same embedding aperture but different hole lengths. The right y-axis represents the SAC, while the left y-axis displays the corresponding resonance frequency. The line chart is used to show the trend of the first and second order resonance frequencies as a function of aperture and hole length. From Fig 10(A), when the aperture was 2mm, the first resonance frequency and absorption peak were 104Hz and 0.98, respectively, and the second resonance frequency and absorption peak were 123Hz and 0.64, respectively. When the aperture was 6mm, the first resonance frequency was 106Hz, the absorption peak was 0.86, the second resonance frequency was 122Hz, and the absorption frequency was 0.70. When the aperture was 10mm, the first resonance peak appeared at 108Hz, the first absorption peak was 0.75, the second resonance peak appeared at 184Hz, and the second absorption peak was 0.81. When the aperture was 16mm, the first resonance frequency generated was 114Hz, the first absorption peak was 0.71, the second resonance frequency was 220Hz, and the second absorption peak was 0.80. As the aperture gradually increased, the first order resonance frequency showed an increasing trend, the first order absorption peak showed a decreasing trend, and the second order resonance frequency and absorption peak both showed an upward trend. From Fig 10(B), when the hole lengths were 20mm, 40mm, 60mm, and 80mm, the first resonance frequency was 108Hz, 106Hz, 105Hz, and 104Hz, and the corresponding absorption peaks for different hole lengths were 0.75, 0.90, 0.96, and 0.99, respectively. The second order resonance frequencies were 185Hz, 140Hz, 139Hz, and 138Hz, respectively, with corresponding absorption peaks of 0.81, 0.70, 0.62, and 0.44. As the hole length increased, the first order resonance frequency gradually decreased, the first order absorption peak gradually increased, and the second order resonance frequency and absorption peak showed a decreasing trend. Setting appropriate embedded aperture and hole length can improve the sound absorption of walls.

### 3.2Analysis of wall acoustic insulation effect based on BIM technology and ITM

**Fig 11:**
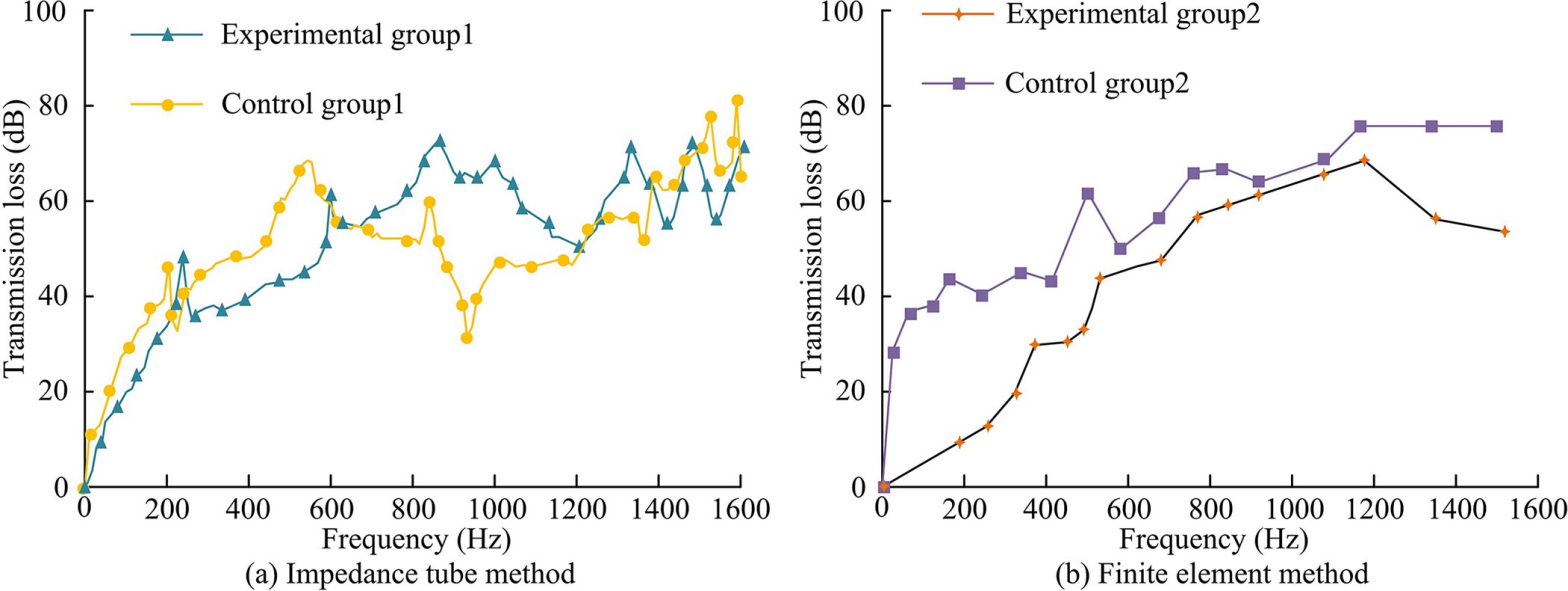
Sound insulation of two sets of specimens at different acoustic frequencies.

To further explore the acoustic performance of WSB walls that integrate BIM technology and ITM, the testing equipment and supporting equipment were kept unchanged. The sound insulation of the two groups was tested at a frequency of 90-1600Hz. Firstly, it calculated the changes in sound insulation performance of two sets of specimens under different acoustic frequencies, and compared them with the sound insulation performance of specimens based on the finite element method. The outcomes are indicated in Fig 11.

**Fig 12:**
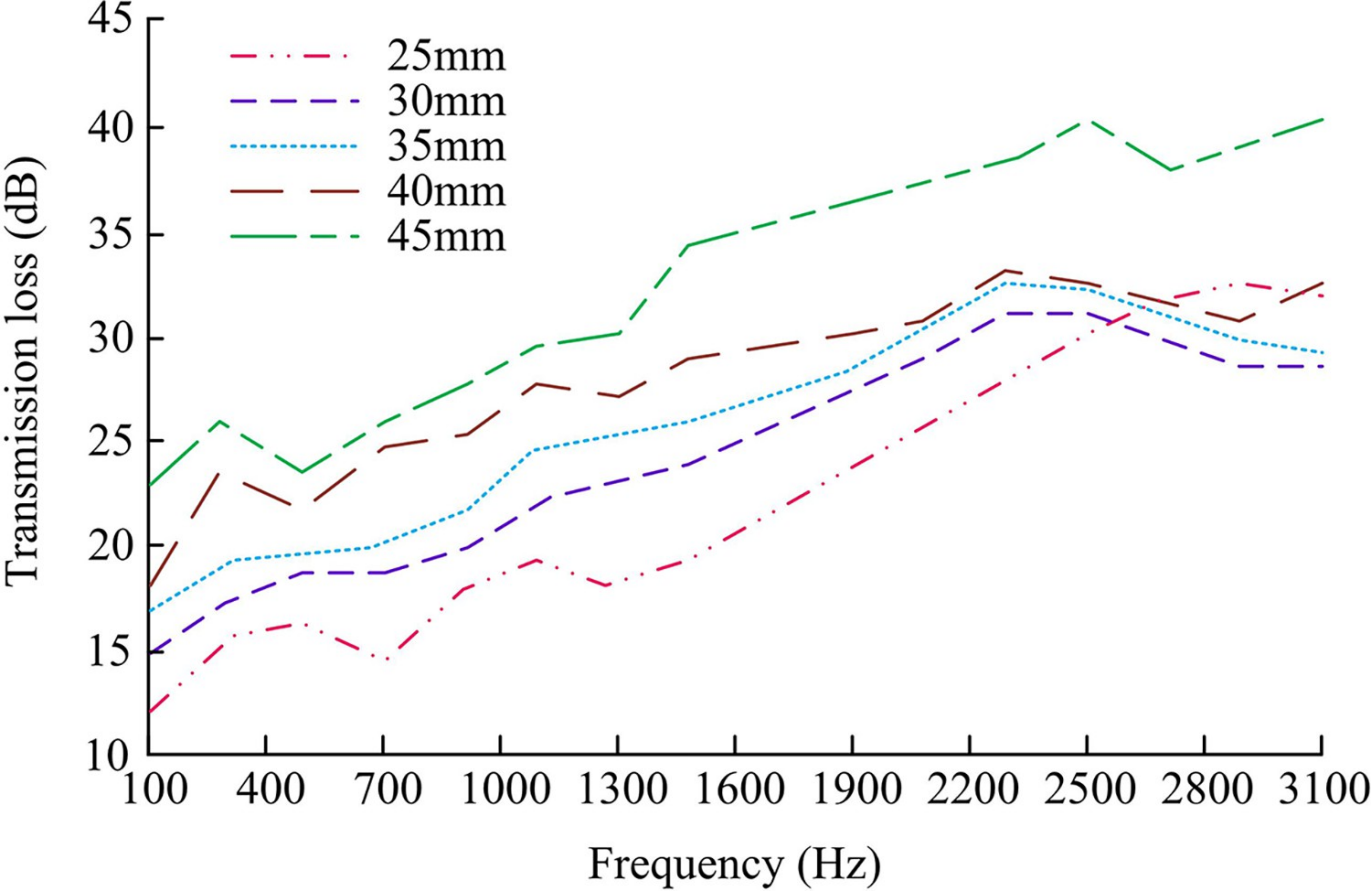
Sound insulation performance of wood structure walls with different thicknesses.

Fig 11(A) and 11(B) show the changes in sound insulation of different specimens at different sound frequencies calculated using design methods and finite element method, respectively. From Fig 11 (A), when the frequency was 500Hz, the sound insulation level of control group 1 was 50.29dB, and the sound insulation level of experimental group 1 was 41.83dB. The difference between the two gradually narrowed. When the frequency was 1250Hz, the sound insulation of control group 1 was 51.26dB, and the sound insulation of experimental group 1 was 51.28dB. When the frequency was 1600Hz, the sound insulation of control group 1 was 65.30dB, and the sound insulation of experimental group 1 was 70.14dB. From Fig 11(B), when the frequency was 500Hz, the sound insulation volume of control group 2 was 31.07dB, and that of experimental group 2 was 44.36dB. When the frequency was 1250Hz, the sound insulation of control group 2 was 63.24dB, and the sound insulation of experimental group was 77.19dB. The variation trend of sound insulation of specimens based on finite element method was consistent with that of specimens based on BIM technology and ITM, indicating that the difference in sound insulation between the two group was not significant. However, compared to the control group, the sound insulation of the experimental group was more stable and could maintain a high sound insulation effect even at higher frequencies. The next step was to test the sound insulation of WSB with different thicknesses, as shown in Fig 12.

**Fig 13:**
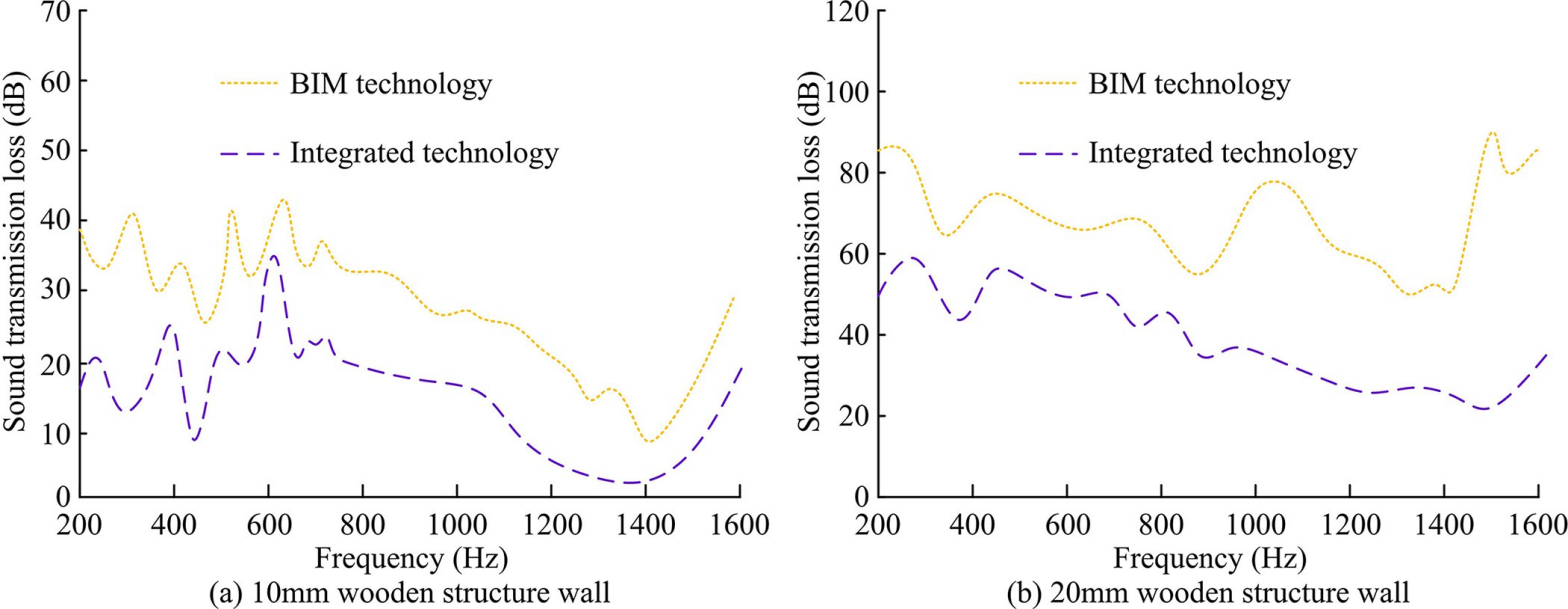
Sound transmission loss under different techniques of 10mm and 20mm thick wooden structure walls.

From Fig 12, in the case of thinner wall thickness, the sound insulation was relatively low. This is because thin walls cannot effectively block the propagation of sound waves, making it easier for sound to pass through the walls. However, as the thickness of the wall increased, the sound insulation gradually increased. This is because thick walls can more effectively absorb and attenuate sound waves, reduce the transmission of sound through the walls, and achieve good sound insulation effects. Meanwhile, as the frequency increased, the sound insulation also showed an increasing trend. High frequency sound waves vibrated faster and had more concentrated energy, making them easier to absorb and attenuate. However, low-frequency sound waves vibrated slower and had more dispersed energy, making it relatively easier to penetrate walls [22]. Therefore, the isolation effect of walls on high-frequency sound was better than that of low-frequency sound. Next, the sound transmission loss of the designed integrated technology and the traditional BIM technology was tested under the 10mm and 20mm thick wooden structure walls, and the results are shown in Fig 13.

**Fig 14:**
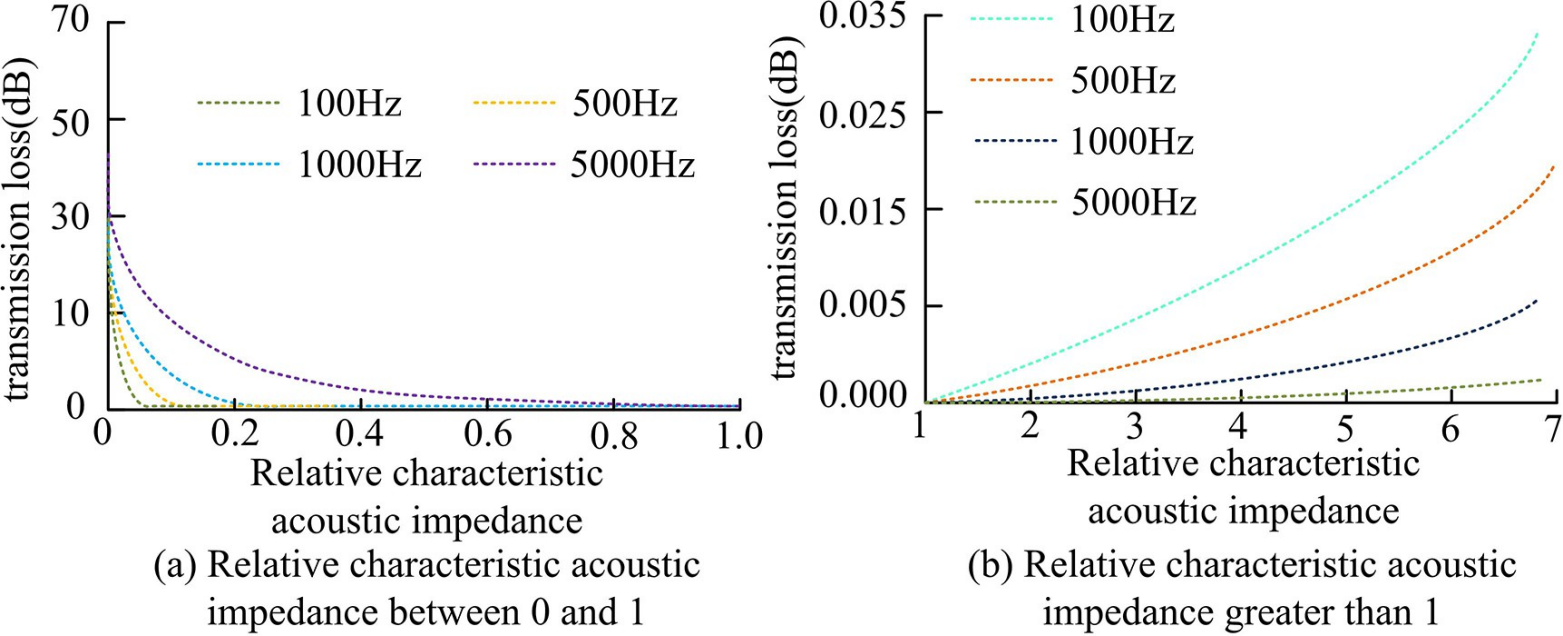
The relationship between relative characteristic AI and sound insulation at different frequencies.

Fig 13(A) and 13(B) show the sound transmission losses of different methods under a 10mm and a 20mm thick wooden structure walls, respectively. From Fig 13(A), under a 10mm thick wooden structure wall, the average sound transmission loss of traditional BIM technology was 32.4dB, and the average sound transmission loss of the designed algorithm was 17.3dB. From Fig 13(B), under a 20mm thick wooden structure wall, the average sound transmission losses of traditional BIM technology and designed algorithm were 73.6dB and 39.7dB, respectively. The average loss of the designed algorithm under different thicknesses of wooden structural walls was about 46% lower than that of traditional BIM technology, further proving its good sound insulation effect. Next, the effect of relative characteristic AI on sound insulation at various frequencies was examined, as denoted in Fig 14.

**Fig 15:**
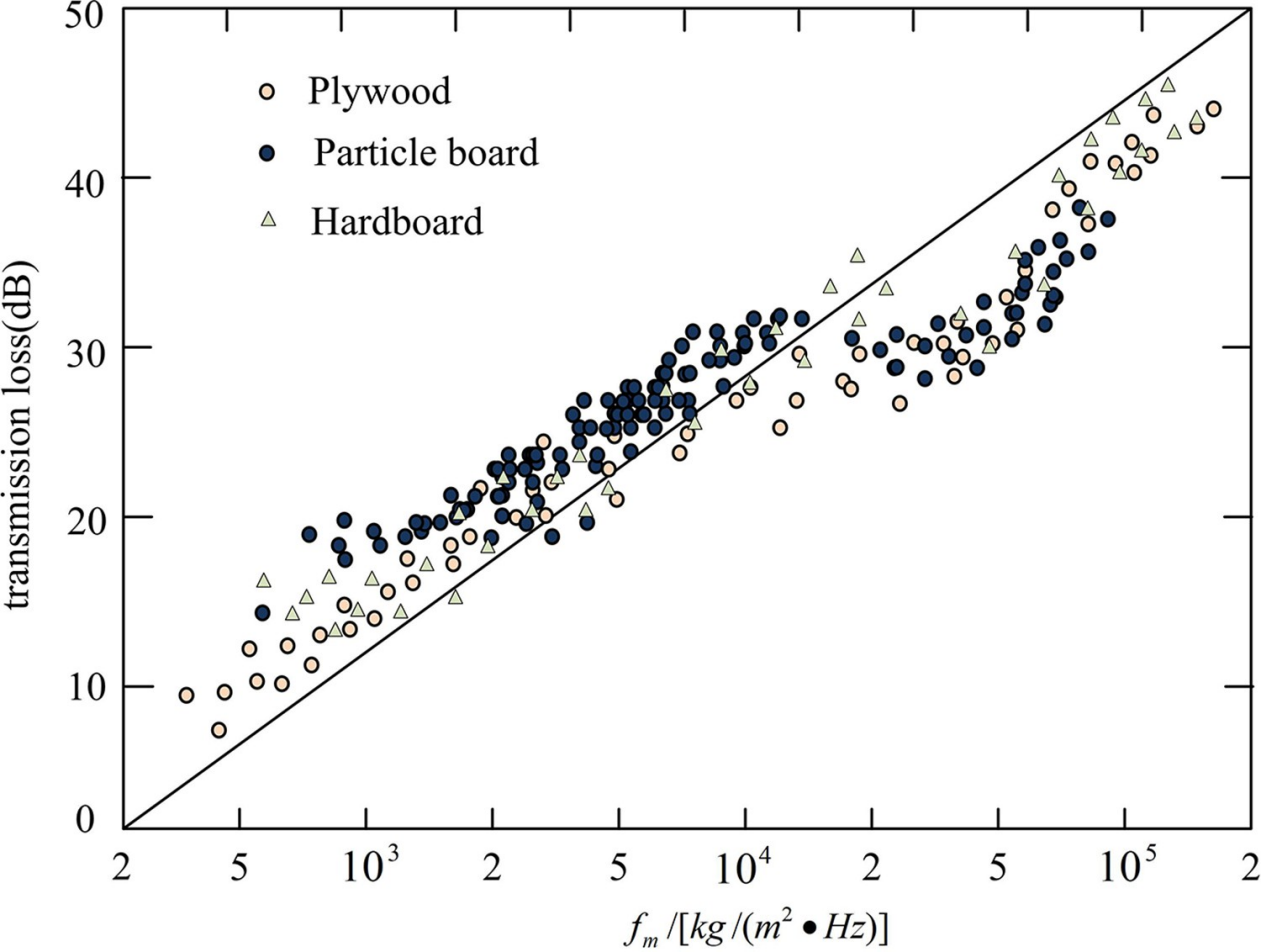
Sound insulation performance of different types of wood structure walls.

Fig 14(A) and 14(B) show the effect of relative characteristic impedance between 0 and 1 and greater than 1 on sound insulation at different frequencies, respectively. From Fig 14(A), when the relative characteristic AI was between 0 and 1, the sound insulation decreased with the increase of the relative characteristic AI. It indicated that when the changes in the propagation medium of sound waves were small, the sound insulation performance would decrease accordingly. This was because sound waves interact with surrounding media during their propagation. When the characteristic impedance of the medium did not match the impedance of the sound waves, sound waves would scatter and attenuate, leading to a decrease in sound insulation performance [23]. From Fig 14(B), when the relative characteristic AI was greater than 1, the sound insulation amount increased with the increase of the relative characteristic AI. It indicated that when there was a significant change in the propagation medium of sound waves, the sound insulation performance would be improved accordingly. This is because when the characteristic impedance of the medium matches the impedance of the sound wave, the sound wave can propagate better, while reducing reflection and scattering, thereby improving sound insulation performance [24]. According to the law of mass, the sound insulation performance of walls is directly proportional to the mass per unit area and is related to the frequency of incident sound waves. The results are shown in Fig 15.

In Fig 15, the x-axis represents the sound insulation performance of the wall, and the y-axis represents the transmission loss. From Fig 15, the sound insulation of different wooden structure walls showed a similar trend with frequency variation, verifying the authenticity of the data. When the frequency was high, the sound insulation of all walls was lower than the standard line, indicating that the sound insulation effect of the wall was poor. This is because high-frequency sound propagation is easier to penetrate walls, thereby reducing the sound insulation effect [25]. When the frequency was low, the sound insulation of all walls was higher than the standard line, indicating that the sound insulation of the wall was good. The effectiveness of combining BIM technology and ITM in wall sound insulation performance testing was proved.

## 4. Discussion

The results of this study indicated that the integration of BIM technology and ITM was effective in evaluating the acoustic performance of WSB, especially in the measurement of sound absorption and insulation performance. Compared with existing research, the proposed method demonstrated high accuracy and reliability in predicting SAC and sound insulation, which was partly attributed to the accuracy of BIM models in simulating building acoustic behavior. In addition, this study verified the optimization of wall unit structure design through experiments, such as the introduction of holes and slots, which had a significant effect on improving the sound absorption performance at medium and low frequencies. This discovery was consistent with the conclusion of Yuvaraj L et al. [3] when studying the acoustic performance of micro perforated panels, that structural adjustment can effectively expand the sound absorption bandwidth. However, in the high frequency range, the effect of the pore groove structure was not as expected, which may point to the direction of future research, that is, how to optimize the absorption of high frequency sound waves by adjusting the pore groove structure or using different material combinations. Meanwhile, the study also found a certain correlation between sound insulation and frequency, that specific design and material selection can significantly improve the sound insulation performance of the structure. Furthermore, the impact of relative AI on sound insulation was also validated in this study, which was also corroborated by the findings of Zamora Mestre et al. [4]. This research can investigate the influence of different material characteristics on the propagation of sound waves and the potential for modifying this parameter through design to enhance sound insulation effects.

Overall, this study not only provides a new methodological framework for testing and evaluating the acoustic performance of WSB, but also provides practical guidance on how to optimize building acoustic performance in the design phase in the future. This has important practical significance for designing a quieter and more comfortable living and working environment.

## 5. Conclusion

With the development of technology and the increasing demand for building environments, the acoustic performance of buildings has become an important factor that designers and engineers must consider. As a traditional architectural form, the research and application of acoustic performance of WSBs are also receiving increasing attention. To better analyze and optimize the acoustic performance of WSBs, the study integrated BIM technology with ITM to explore the optimization path of acoustic performance of wooden structures, filling the research gap in this field. By constructing a building performance testing system based on BIM technology and applying ITM to it, the performance of wall buildings was tested. The outcomes indicated that in the variation curves of the SAC under different sound wave frequencies of the two groups of specimens, when the sound wave frequency was 185Hz, the SAC of the control group was 0.01, while the SAC of the experimental group was 0.81. This indicated the effectiveness of integrating BIM technology with ITM for building sound absorption testing. In the error analysis between the two groups, the average error was 0.097, and the difference between the two groups was small, indicating that the integration of BIM technology and ITM had high reliability. In the detection of sound insulation performance of different types of wooden structure walls, when the frequency was high, the sound insulation effect was poor, while when the frequency was low, the sound insulation was good, proving the effectiveness of the designed algorithm. This technology enables designers to extract the requisite building information and optimize the WSB based on experimental results, software simulations, and on-site measurements. This process effectively improves the acoustic environment of the WSB and enhances the overall acoustic performance of the building. Consequently, it provides technical support for sound absorption and noise reduction of building walls.

## 6. Limitations

Although research has demonstrated the effectiveness of the integration technology of BIM and impedance tube, the evaluation scope of acoustic performance is not fully covered. For instance, the scattering, diffraction, and reflection phenomena of sound waves in complex building environments can render the propagation path of sound more intricate, thereby influencing the acoustic performance of walls. The acoustic performance of a wall may also be influenced by the intrinsic properties of the material used. For instance, the anisotropy of wood can affect the accuracy of acoustic analysis, as can the precision of the physical parameter values associated with it. In the future, the exploration of the propagation mechanism of sound waves will be strengthened, including the interaction between the internal and external sound fields of buildings, the optimization of acoustic performance of various building materials, and the adaptability of acoustic models in diverse application environments. At the same time, a large number of experimental tests can be conducted on the main physical parameters of commonly used structural materials, and records can be organized into a database to improve the accuracy and practicality of acoustic performance. Field experiments and measurements can also be added to measure and determine the specific optimization indicators and directions of the building and its wall structure.

## Supporting information

S1 Data set (DOCX)
