# Supplementary material for: Acoustic performance analysis of wooden structure building wall by integrating BIM technology and impedance tube method
Source: PLoS One. 2024 Aug 9;19(8):e0308481. doi: 10.1371/journal.pone.0308481 (PMC11315332; doi:10.1371/journal.pone.0308481)
Supplement: S1 Data set — (DOCX) [file pone.0308481.s001.docx]

The Data in Figure 8

| Frequency (Hz) | | | 200 | 250 | 400 | 600 | 800 | 1000 | 1200 | 1400 | 1600 |
| --- | --- | --- | --- | --- | --- | --- | --- | --- | --- | --- | --- |
| Sound absorption coefficient (dB) | Method | OSB panel material thickness (mm) |  |  |  |  |  |  |  |  |  |
|  | Impedance tube method | 10 | 0.046 | 0.048 | 0.051 | 0.072 | 0.083 | 0.093 | 0.085 | 0.541 | 0.182 |
|  |  | 20 | 0.037 | 0.042 | 0.035 | 0.046 | 0.069 | 0.093 | 0.078 | 0.204 | 0.147 |
|  | Finite element method | 10 | 0.046 | 0.048 | 0.042 | 0.047 | 0.074 | 0.116 | 0.428 | 0.352 | 0.037 |
|  |  | 20 | 0.039 | 0.037 | 0.033 | 0.041 | 0.043 | 0.084 | 0.194 | 0.193 | 0.179 |

The Data in Figure 9

| Frequency (Hz) | | 100 | 110 | 185 | 200 | 400 | 600 | 800 | 1000 | 1200 | 1365 | 1400 | 1600 |
| --- | --- | --- | --- | --- | --- | --- | --- | --- | --- | --- | --- | --- | --- |
| Sound absorption coefficient (dB) | Experimental group | 0.50 | 0.68 | 0.81 | 0.96 | 0.40 | 0.24 | 0.18 | 0.14 | 0.13 | 0.11 | 0.12 | 0.11 |
|  | Control group | 0.08 | 0.07 | 0.01 | 0.08 | 0.09 | 0.05 | 0.03 | 0.03 | 0.07 | 0.25 | 0.23 | 0.11 |

The Data in Figure 10

| Test-piece | | Resonance frequency(Hz) | | Sound absorption coefficient | |
| --- | --- | --- | --- | --- | --- |
|  |  | First-order | Second-order | First-order | Second-order |
| Same hole length but different aperture | K10-L20 | 104 | 123 | 0.98 | 0.64 |
|  | K06-L20 | 106 | 122 | 0.86 | 0.70 |
|  | K10-L20 | 108 | 184 | 0.75 | 0.81 |
|  | K10-L80 | 114 | 220 | 0.71 | 0.80 |
| Same aperture but different hole lengths | K10-L20 | 108 | 0.75 | 185 | 0.81 |
|  | K10-L40 | 106 | 0.90 | 140 | 0.70 |
|  | K10-L60 | 105 | 0.96 | 139 | 0.62 |
|  | K10-L80 | 104 | 0.99 | 138 | 0.44 |

The Data in Figure 11

| Frequency (Hz) | | | 0 | 200 | 400 | 500 | 600 | 800 | 1000 | 1200 | 1250 | 1400 | 1600 |
| --- | --- | --- | --- | --- | --- | --- | --- | --- | --- | --- | --- | --- | --- |
| Transmission loss (dB) | Method | Test-piece |  |  |  |  |  |  |  |  |  |  |  |
|  | Impedance tube method | Experimental group1 | 0 | 36.74 | 39.41 | 41.83 | 61.59 | 63.47 | 67.44 | 51.16 | 51.28 | 60.03 | 70.14 |
|  |  | Control group1 | 0 | 46.32 | 48.65 | 50.29 | 59.92 | 52.76 | 46.37 | 48.24 | 51.26 | 64.87 | 65.30 |
|  | Finite element method | Experimental group2 | 0 | 10.13 | 31.42 | 44.36 | 46.74 | 58.16 | 63.51 | 66.83 | 77.19 | 77.63 | 78.14 |
|  |  | Control group2 | 0 | 42.36 | 43.81 | 31.07 | 52.71 | 66.43 | 67.38 | 66.15 | 63.24 | 55.26 | 53.68 |

The Data in Figure12

| Transmission loss (dB) | | 100 | 400 | 700 | 1000 | 1300 | 1600 | 1900 | 2200 | 2500 | 2800 | 3100 |
| --- | --- | --- | --- | --- | --- | --- | --- | --- | --- | --- | --- | --- |
| Thickness of wooden building walls (mm) | 25 | 12.1 | 16.7 | 14.5 | 18.4 | 18.2 | 20.4 | 20.6 | 27.0 | 30.1 | 32.1 | 32.0 |
|  | 30 | 14.9 | 18.1 | 18.8 | 20.9 | 23.0 | 24.9 | 27.6 | 30.1 | 31.0 | 29.0 | 28.7 |
|  | 35 | 16.8 | 19.5 | 20.1 | 23.0 | 25.2 | 26.5 | 28.4 | 31.9 | 32.2 | 30.6 | 29.4 |
|  | 40 | 18.0 | 22.4 | 23.1 | 26.3 | 27.1 | 29.4 | 30.1 | 32.1 | 32.9 | 31.5 | 32.9 |
|  | 45 | 22.9 | 24.7 | 26.0 | 28.8 | 30.1 | 35.0 | 36.4 | 38.0 | 40.2 | 38.6 | 40.4 |

The Data in Figure 13

| Frequency (Hz) | | | 200 | 400 | 600 | 800 | 1000 | 1200 | 1400 | 1600 |
| --- | --- | --- | --- | --- | --- | --- | --- | --- | --- | --- |
| Sound transmission loss (dB) | Thickness of wooden wall structure | Test-piece |  |  |  |  |  |  |  |  |
|  | 10mm wooden structure wall | BIM technology | 38.9 | 33.0 | 37.7 | 32.6 | 27.3 | 21.5 | 8.9 | 30.0 |
|  |  | Integrated technology | 16.6 | 23.9 | 32.8 | 19.3 | 17.2 | 5.9 | 2.2 | 28.7 |
|  | 20mm wooden structure wall | BIM technology | 85.4 | 70.5 | 66.3 | 64.0 | 75.9 | 60.0 | 51.3 | 85.7 |
|  |  | Integrated technology | 45.7 | 47.6 | 49.9 | 45.2 | 36.0 | 27.3 | 26.1 | 33.4 |

The Data in Figure 14 (a)

| Relative characteristic acoustic impedance | | 0 | 0.2 | 0.4 | 0.6 | 0.8 | 1.0 |
| --- | --- | --- | --- | --- | --- | --- | --- |
| transmission loss(dB) | 100Hz | 30.0 | 0.0 | 0.0 | 0.0 | 0.0 | 0.0 |
|  | 500Hz | 30.0 | 0.0 | 0.0 | 0.0 | 0.0 | 0.0 |
|  | 1000Hz | 30.0 | 2.0 | 0.0 | 0.0 | 0.0 | 0.0 |
|  | 5000Hz | 40.0 | 5.0 | 3.0 | 2.0 | 1.0 | 0.0 |

The Data in Figure 14 (b)

| Relative characteristic acoustic impedance | | 1 | 2 | 3 | 4 | 5 | 6 | 7 |
| --- | --- | --- | --- | --- | --- | --- | --- | --- |
| transmission loss(dB) | 100Hz | 0.000 | 0.000 | 0.000 | 0.0005 | 0.0007 | 0.001 | 0.015 |
|  | 500Hz | 0.000 | 0.000 | 0.0005 | 0.001 | 0.002 | 0.0035 | 0.006 |
|  | 1000Hz | 0.000 | 0.001 | 0.002 | 0.0035 | 0.0055 | 0.008 | 0.0125 |
|  | 5000Hz | 0.000 | 0.002 | 0.0045 | 0.007 | 0.01 | 0.014 | 0.02 |

The Data in Figure 15

| Type | Plate thickness | Area density | Transmission loss | | | | | | |
| --- | --- | --- | --- | --- | --- | --- | --- | --- | --- |
|  |  |  | 125Hz | 250Hz | 500Hz | 1000Hz | 2000Hz | 4000Hz | Average value |
| Plywood | 6.0 | 3.0 | 11.0 | 13.0 | 16.0 | 21.0 | 25.0 | 23.0 | 18.2 |
|  | 120 | 8.0 | 18.0 | 20.0 | 24.0 | 24.0 | 25.0 | 30.0 | 23.5 |
|  | 40.0 | 24.0 | 24.0 | 25.0 | 27.0 | 30.0 | 38.0 | 43.0 | 31.2 |
| Particle board | 6.0 | 4.6 | 18.0 | 18.0 | 22.0 | 27.0 | 32.0 | 31.0 | 24.7 |
|  | 20.0 | 13.0 | 24.0 | 27.0 | 26.0 | 27.0 | 24.0 | 33.0 | 26.8 |
|  | 35.0 | 17.0 | 21.0 | 23.0 | 27.0 | 28.0 | 24.0 | 29.0 | 25.3 |
| Hardboard | 5.0 | 5.2 | 21.0 | 21.0 | 23.0 | 27.0 | 33.0 | 36.0 | 26.8 |
